# Workplace mental wellbeing interventions in the construction industry: a systematic review of outcome reporting as a first step for the development of a core outcomes set

**DOI:** 10.1186/s12889-026-28427-5

**Published:** 2026-07-16

**Authors:** Chloe Mitchell, David Nickson, Amber F. Tout, Richard Cooke, Rachel Povey, Kevin Daniels, Kirstie Haywood, Carla T. Toro

**Affiliations:** 1 Warwick Medical School, Warwick Applied Health, Mental Health Innovations Group, University of Warwick, Coventry, CV4 7HL UK; 2 Psychology Department, School of Health, Education, Policing and Sciences, Science Centre, University of Staffordshire, Stoke On Trent, ST4 2DF UK; 3 Norwich Business School, University of East Anglia, Norwich, NR4 7TJ UK; 4 Warwick Medical School, Warwick Applied Health, University of Warwick, Coventry, CV4 7HL UK

**Keywords:** Mental wellbeing, Construction industry, Outcome reporting, Systematic review, Workforce, Workplace, Psychosocial stressor

## Abstract

**Background:**

Construction workers experience high job demands and challenging work conditions; therefore, evidence-based interventions to support the wellbeing of workers are needed. This systematic review describes previous studies that have tested interventions to improve the mental wellbeing of construction workers. Outcome measures used within the studies were summarised as a first step in developing a Core Outcome Set (COS) for workplace mental wellbeing interventions in the construction industry.

**Methods:**

The NICE NG212 Mental Wellbeing at Work guideline definition and categorisation of mental wellbeing interventions was used to guide search terms to identify intervention studies to improve construction worker mental wellbeing in four online databases: PsycINFO, Web of Science, MEDLINE and Scopus. Intervention study type and participant characteristics were summarised, followed by patterns of outcome reporting with outcomes inductively mapped to outcome domains.

**Results:**

Sixteen studies from eight countries were included. A large proportion (7 of 16) evaluated components of the Australian MATES suicide prevention programme or an Australian electronic mental health stigma intervention. Other interventions were organisational or targeted supervisors, peers and/or individual workers. A total of 43 mental wellbeing relevant outcomes were reported and these were inductively categorised into four domains: (1) mental health and wellbeing, (2) work processes and environment, (3) mental health stigma, behaviours, or attitudes, and (4) suicide awareness, knowledge, beliefs, or behaviours. The greatest number of outcomes fell into domain 2 (16/45; 37%). The seven Australian studies reported all outcomes for two of the outcome domains (domains (3) and (4)). No economic outcomes were reported in any of the studies.

**Conclusions:**

A variety of organisational or supervisor, peer and/or individual targeted interventions to improve mental wellbeing of construction workers have been evaluated. Outcome reporting related to mental wellbeing fell into four broad outcome domains, with substantial heterogeneity within these categories. This provides an important first step for the development of a COS to enable researchers and organisations to use a consistent approach to data collection and evaluate interventions to improve mental wellbeing for the construction workforce.

**Trial registration:**

Research England Business and Commercialisation Fund. International Prospective Register of Systematic Reviews (PROSPERO): CRD42023410717.

**Supplementary Information:**

The online version contains supplementary material available at https://doi.org/10.1186/s12889-026-28427-5.

## Introduction

Academic and industry body-led research has consistently highlighted high levels of poor mental wellbeing for construction workers [1–3]. For example, a recent 2025 survey by the Chartered Institute of Building (CIOB) identified that out of 865 construction workers, in the past year, 83% had experienced anxiety, 60% had experienced depression, 94% had experienced stress and 27% had had suicidal thoughts [4]. Burnout has also been highlighted as prevalent amongst construction workers [5, 6], and some studies cite increased drug and alcohol mis-use as a consequence of poor mental wellbeing [7]. Concerningly, there are also high suicide rates among construction workers compared to other occupations and the wider population [8–12]. Accordingly, mental ‘ill-health’ has been described as the ‘silent crisis within the construction industry’ [3].

There are distinctive occupational challenges faced by construction workers that may adversely impact mental wellbeing; research suggest that many of these challenges relate to the temporary and resource constrained nature of construction projects [3, 13–15]. Office-based professional workers report experiencing stress related to industry competition and time pressures, whereas on-the-tools trade workers report high job demands, excessive work hours and poor social support [16, 17]. Across all industries in the UK, construction has the largest proportion of workers who are self-employed (36% of the workforce) [18] and the highest proportion of small or medium-sized enterprises (SMEs, organizations with fewer than 250 employees [19]) than other industries [20]. These features are associated with more severe financial insecurity and less initiatives to support mental wellbeing [21]. There are parallels in rates of self-employment with other counties such as USA (with 22% self-employed; [22] and Australia [23]. Suicide rates are significantly higher in construction than other industries across these 3 countries; in the UK suicide rates are 3 times higher [12], in Australia they are 2 times higher [10] and in USA 2.4 times higher [9]. The construction industry is male dominated, with men making up 85.3% of the workforce within the UK [24], contributing to a macho culture and barriers to help-seeking [25].

In light of the growing evidence for poor mental wellbeing among construction workers, there have been repeated calls for the construction industry to improve mental wellbeing support for its workers (e.g., [26, 27]). In the UK, construction firms commonly rely on toolbox talks, Mental Health First Aid (MHFA) training and helplines [28]. Despite these efforts, research led by a UK industry body identified a lack of a “dashboard” for measurement and assessment of effectiveness of these interventions and concurrently, low practice in evaluating effectiveness of interventions [28]. In recognition of this problem across sectors, the committee behind the National Institute for Health and Care Excellence (NICE) NG212 Workplace Mental Wellbeing guidance proposed a future research priority that standardised guidance for routine monitoring and assessing effectiveness of interventions for mental wellbeing at work is developed [29]. The NICE committee recommended that this priority is addressed using Core Outcome Set (COS) methodology [29].

COS research, originally established in clinical sciences, seeks to define a group of outcomes or factors that should be measured across all effectiveness trials or for routine assessment [30]. This offers the potential to tackle three prevailing issues in health intervention and routine monitoring studies: (i) heterogeneity in outcome reporting leading to limitations in evidence syntheses [31]; (ii) outcome reporting bias [32]; (iii) limited consideration of outcomes important to patients [33]. More recent COS have been developed for public health and discrete examples of wellbeing at work such as return to work [34] and doctor wellbeing [35].

Guidance for COS development is well-established [36] and there is also an international database of all previous and ongoing COS research [37]. The guidance specifies four broad areas of activities for the development of a new COS and the establishment of a COS Working Group of experts to support decision-making during COS development. The first phase is to establish the scope of the COS i.e., the population, setting, types of interventions or routine monitoring needs. This may not conclude until the second phase is completed which involves conducting reviews of the literature on what outcomes or factors have been measured previously. The outputs of the reviews result in the generation of a long-list of outcomes for consideration by the COS Working Group before entering the third phase which involves a multistep consensus and prioritisation process with key stakeholders to identify which outcomes should be included in the COS.

In line with best practice recommendations for COS development [30, 38, 39], this review will identify published workplace wellbeing intervention studies in the construction industry to characterise all outcomes that have been assessed previously.

## Methods

This systematic review was conducted and reported in accordance with the Preferred Reporting Items for Systematic Reviews and Meta Analyses (PRISMA) guidelines [40]. The protocol is registered with the International Prospective Register of Systematic Reviews (PROSPERO): CRD42023410717.

### Search strategy

Through consultation with an expert librarian (SJ, University of Warwick), a comprehensive search strategy was compiled to identify all studies evaluating an intervention designed to target mental wellbeing within the construction industry. The search terms included synonyms of; 1) construction workforce, 2) mental wellbeing, 3) interventions and 4) workplace (detailed in supplementary file 1).

Four online databases were searched (prior to August 2023 and, as an update, to end of January 2025): PsycINFO, Web of Science, MEDLINE and Scopus. These databases were selected as they index a range of wellbeing and construction related journals. Reference lists of systematic reviews identified as relevant to the topic area were hand searched to identify any articles that had been missed via our own searches.

### Eligibility criteria for screening

Included studies reported quantitative assessments of interventions designed to improve construction worker mental wellbeing (see Table 1). Construction industry workers were defined as: any persons engaged in the construction industry as professionals, supervisors, construction project managers engaged in planning, coordinating, and controlling construction projects, and frontline workers involved in manual aspects of construction work [13].

**Table 1:** Study eligibility criteria for screening

	**Inclusion**	**Exclusion**
Participants	Primary population of employees aged 16 and over that work within the construction industry. This can include individuals that are: • Self-employed • Work at/for micro, small and medium enterprises (SME’s) in addition to larger national/international level construction businesses • Employees in full or part time work including those in permanent, training, temporary or zero-hour contracts	Child workers under the age of 16 Studies that do not focus on workers within the construction industry as the primary population
Intervention	Interventions designed to improve mental wellbeing within the construction workforce	Interventions that were not designed to improve mental wellbeing within the construction workforce
Setting	Any intervention that is delivered within the workplace, or delivered outside of the workplace but was designed, delivered, managed, funded or signposted to by the employer, delivered in person or delivered/accessed online/digitally	Studies based on national public health campaigns
Study Design	Quantitative Pre and post intervention measures as a minimum	Qualitative Cross sectional designs, case studies and observational studies, protocols

Mental Wellbeing interventions were defined as per the NICE Mental Wellbeing at Work Evidence Review F [41], to include any interventions that aim to (one or more of):

- Improve mental wellbeing.
- Promote positive mental wellbeing.
- Prevent poor mental wellbeing.
- Increase awareness or understanding of mental wellbeing.
- Help managers understand, recognise, and respond to their employee’s mental wellbeing.

Both interventions with a focus on improving mental wellbeing directly (e.g., psychological therapy) and indirectly (e.g., via the work environment) were appropriate for inclusion. Included studies also needed to be published in peer-reviewed academic journals and available as a full-text article written in English.

### Study selection

Abstracts were retrieved from the databases and uploaded to Rayyan [42] systematic review software. All titles and abstracts were reviewed for inclusion/exclusion by one author (Aug 2023). A randomly assigned 10% subset were screened by a second author with 100% agreement achieved. This process was repeated for the full texts. There was one disagreement which was discussed with a third author who provided the deciding vote for inclusion. This was repeated for the update search (Jan 2025) where there were no disagreements.

### Data extraction

Data were extracted into a predefined template (Excel; Microsoft, USA). Study specific information included: author, year of publication, country, study design, study population (number of participants, demographics, participant job role), size of organisation, study aims and intervention design, effectiveness for outcomes relevant to mental wellbeing.

Data relevant to the interventions was extracted including: the rationale behind the intervention, the study aims, study design, sample size, demographic characteristics of the sample, focus on professional and/or trade construction workers and effectiveness in relation to mental wellbeing.

For development of a COS, outcomes relevant to mental wellbeing were also extracted from all studies. This information was extracted verbatim to capture the assessment focus (what was assessed); method (e.g., which outcome measurement instruments (OMIs) were used; who completed the assessment); and the assessment timing. Where necessary, study authors were contacted to provide missing information.

Apart from the outcome data which was independently/double extracted from all studies by two authors (CM and CT), one author extracted study data (CM initially, then DN for 2025 update); whilst a second author double extracted study data from a 10% sub-set (CT). Double-extracted data was discussed and adapted until agreement was reached.

### Data analysis

Intervention-related data was summarised in table format. Patterns of outcome reporting, including the number of studies reporting the same outcome, frequency of outcome reporting (i.e., what was assessed and how often), and assessment timings were reported using descriptive statistics. An assessment of the conceptual similarity of outcomes supported categorisation into distinct outcome domains. These outcome domains were discussed amongst authors until a consensus was reached.

## Results

### Flow of included studies

**Fig. 1:**
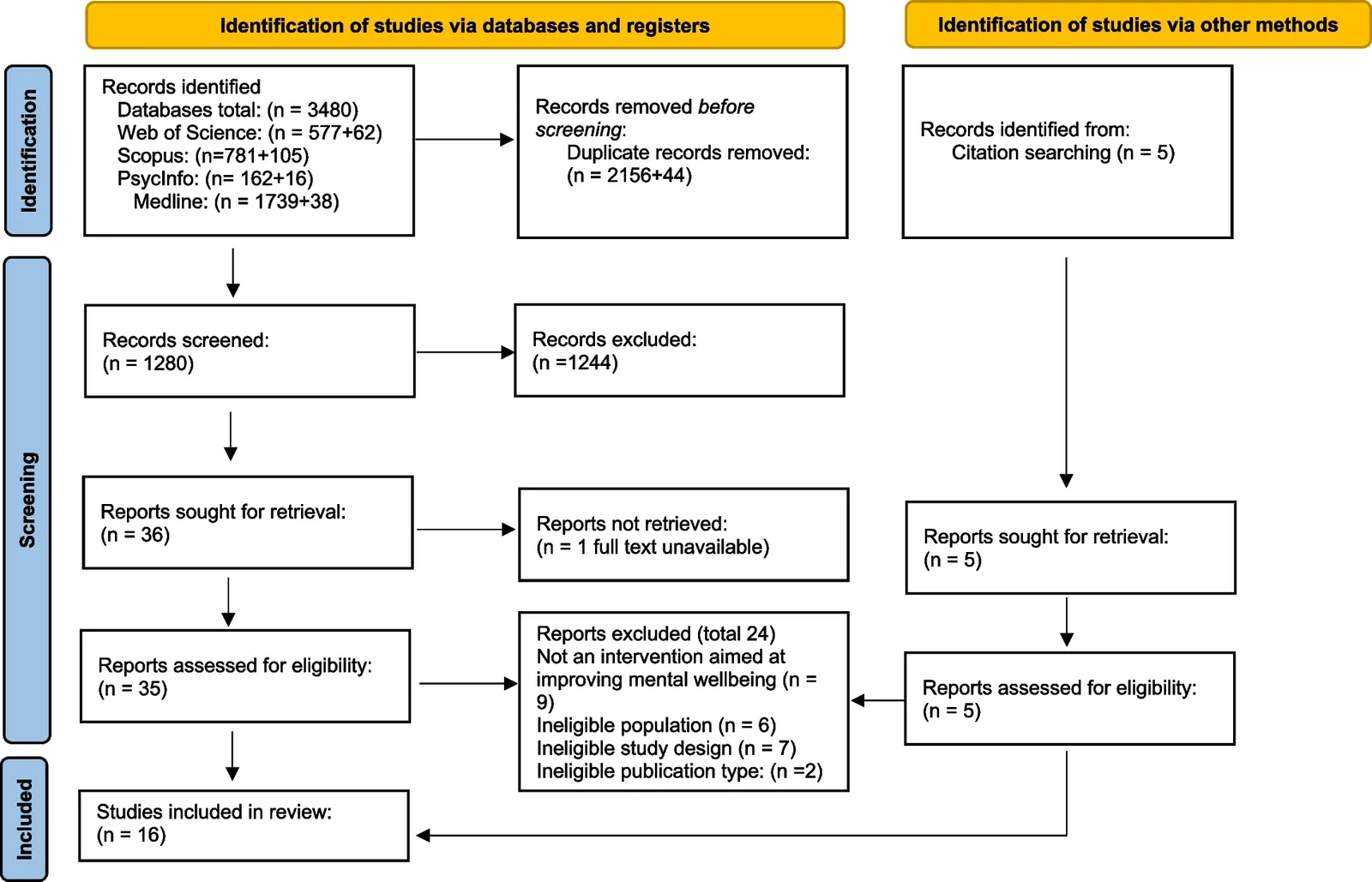
PRISMA Flow chart. *Note: The figures represent the combination of two searches – the original covering to 31/08/23 and an update covering 01/09/23 to 30/01/25 The additional papers are shown for each database. From:* Page MJ, McKenzie JE, Bossuyt PM, Boutron I, Hoffmann TC, Mulrow CD, et al. The PRISMA 2020 statement: an updated guideline for reporting systematic reviews. BMJ 2021;372:n71. https://doi.org/10.1136/bmj.n71. For more information, visit: http://www.prisma-statement.org/

Database searching yielded 3480 hits (Fig. 1). After removal of duplicates (*n* = 2,200), 1,280 studies remained for title/abstract screening; 1244 studies were excluded, and 36 full texts sought for full-text assessment (one of which could not be obtained); 24 studies were subsequently excluded for the following reasons: not an intervention aimed at improving mental wellbeing *n* = 9, ineligible population n = 6 ineligible study design *n* = 7, ineligible publication type *n* = 2. Five additional studies were identified by handsearching the reference list of systematic reviews evaluating construction workplace wellbeing interventions [7, 43, 44] and an intervention study that met eligibility criteria [45]. In total, 16 articles were included [6, 45–54].

### Study and participant characteristics

Intervention studies were conducted across eight countries, with most in Australia (*n* = 7) [49–52, 55–57], three in Nigeria [53, 58, 59], and one each in the United States [46], Sweden [47], Finland [48], Singapore [60], China [45] and Hong Kong [6]. See Supplementary file 2 for a summary of study design and participant characteristics.

Just under half of the studies were randomised controlled trials (RCTs) (*n* = 7/16); two non-randomised controlled trials (12.5%), six before and after studies without a control group (37.5%), and one non-equivalent comparison study (Table 2). Participant numbers ranged from 35 to 20,125 and participants were predominately male, ranging from 69 to 100% male within the studies that reported gender; five studies did not report the gender of participants. Only 3 out of the 16 studies (19%) reported the ethnic background of participants [49, 58, 60], see Supplementary file 2.

**Table 2:** A summary of the aims and effectiveness of the 16 intervention studies

First Author and year	Rationale and Aims of study	Effectiveness (relevant to mental wellbeing)
**Organisational interventions**		
Cedstrand 2022 [47]	Attending to psychosocial stressors: low job control, low role clarity, intervention co-created with construction workers to enhance planning, role and duties clarification and assess impacts on stress and psychosocial conditions	No effects on the primary outcome stress. All outcomes, except role clarity deteriorated during the trial in both intervention and control group. Roll out during the pandemic impacted implementation
Gullestrup 2011 [55]	To assess the impact of mental health and suicide awareness interventions designed by MATES*	Preliminary evidence for intervention effectiveness for improving suicide and mental health awareness, help-seeking behaviour, and treatment engagement
King 2018 [50]	Due to high prevalence of suicide in manual workers, the aims were to explore differences in impacts of MATES* General Awareness Training (GAT) in manual versus professional construction workers	Some short-term impacts on effectiveness. Also, managers and professionals showed greater propensity to shift beliefs, compared to labourers and machinery operators and drivers showed least
King 2019 [56]	Amongst the MATES programme, interventions to improve suicide prevention literacy had been previously tested. Using secondary data, the aims were to explore age differences in beliefs related to suicide prevention and attitudes to the workplace in addressing mental health	The study found that youngest respondents demonstrated the greatest intervention-associated change (higher scores indicating more favourable belief change) (predicted mean belief change 0.47, 95% CI 0.43, 0.50 for those aged 15–24 years compared to 0.38, 95% CI 0.36, 0.41 for men aged 45 years and over)
King 2023 [49]	The study aimed to compare the impacts of two versions of a MATES suicide awareness training on help-seeking intentions	The MATES mobile application, together with face-to-face training, enhanced help-seeking intentions (from a MATES worker/Connector or workmate) to a greater extent than face-to-face training only (p < 0.005)
Milner 2018 [51]	To test if high levels of self-stigma are associated with outcomes, the study aimed to test the effects of an electronic mental health stigma reduction intervention on self-stigma	No significant effect on self-stigma were found compared to control, although participants reported that it was beneficial to their mental health
Milner 2020 [52]	To test if mental health stigma impacts suicidality, the aims were to test the effectiveness of an electronic mental health stigma intervention on suicide ideation, communication about suicide and attempts	The intervention had no significant impact on suicidal thoughts, communication or suicide attempts. There was some indication that individuals in the intervention group reported a slight increase in communication about suicide
Ross 2020 [57]	A longitudinal study to compare the impacts of two versions of a MATES* suicide awareness training on mental health and suicide awareness, help-offering and help-seeking in construction workers	There was no difference between the two interventions and both impacted help-seeking and suicide awareness (p < 0.001)
Yang 2023 [45]	Job crafting has been shown to improve wellbeing for workers in different industries. The aims of this 10-week RCT was to test the effectiveness of the intervention on job crafting behaviours, burnout and work engagement amongst construction project managers	The intervention significantly increased job crafting behaviours, such as increasing social resources (p < 0.01) and decreased burnout (cynicism sub-scale, p < 0.05)
Yip and Rowlinson, 2009 [6]	Job crafting has been shown to improve wellbeing for workers in different industries. The aims of the job crafting study was to assess impacts on burnout of construction professionals one year later	A significant reduction in emotional exhaustion (p < 0.001) and cynicism (p < 0.01) was found
**Supervisor relationships and team interventions**		
Anger 2018 [46]	A holistic training package (Total Worker Health® training) offering supervisor training to increase the frequency of interactions in which supervisors show support for their employees. Also, physical health, nutrition and team building with potential benefits to physical health and team relationships (work climate) in construction setting	The intervention led to significant (p < 0.05) improvements in family-supportive supervisory behaviours (d = 0.72), team cohesion (d = 0.38), sleep duration (d = 0.38), and objectively measured systolic blood pressure (d = 0.27)
Elo 2014 [48]	Based on evidence that leadership behaviours impact wellbeing of direct reports, the aims were to evaluate a 7.5-day personal growth-orientated leadership intervention to supervisors and assess subordinate well-being at work	The authors reported no measurable wellbeing effect. The subordinates’ perception of leadership or of their own well-being did not improve compared to the control group
Palaniappan 2022 [60]	To train migrant workers in Singapore (trained by counsellors in their own mother tongue) to provide peer support to their co-workers for depression, anxiety or stress	Statistically significant (p < 0.05) reductions in mean depression, anxiety and stress scores were recorded following the 6-month intervention
**Individual (targeted) interventions**		
Iremeka 2021 [58]	Rational Emotive Behavioural Therapy (REBT) has evidence of efficacy to reduce poor mental health. The study aimed to ascertain the efficacy of REBT in reducing stress amongst skilled construction workers	REBT significantly reduced stress and work-related irrational beliefs among the skilled construction workers relative to controls (p < 0.05)
Okereke 2022 [59]	Rational Emotive Behavioural Therapy (REBT) has evidence of efficacy to reduce poor mental health. The RCT study aimed to ascertain the efficacy of REBT in reducing burnout among building construction and mechanical trade workers	A significant reduction in mean burnout among workers in the treatment group after the 10-week REBT program relative to the control groups (p < 0.05). This reduction remained at follow-up (p < 0.05)
Omeje 2021 [53]	Based on evidence that CBT can reduce self-reported stress for workers the aim of this pilot RCT were to assess the effects of CBT on work-stress for construction workers in Nigeria	Relative to the waitlist control group, the CBT intervention reduced occupational stress symptoms and dysfunctional thoughts among Nigerian construction trade workers (p < 0.05)

^*^ Studies evaluating elements of the MATES multimodal suicide prevention programme

Occupation was reported in 11 studies. Professional workers in construction (such as project managers) were the focus of two studies [6, 45], while manual (trade) workers were the focus of three studies, all in Nigeria [53, 58, 59]. Six interventions were offered to both trade and professional workers [49–52, 55–57].

### Intervention types

**Fig. 2:**
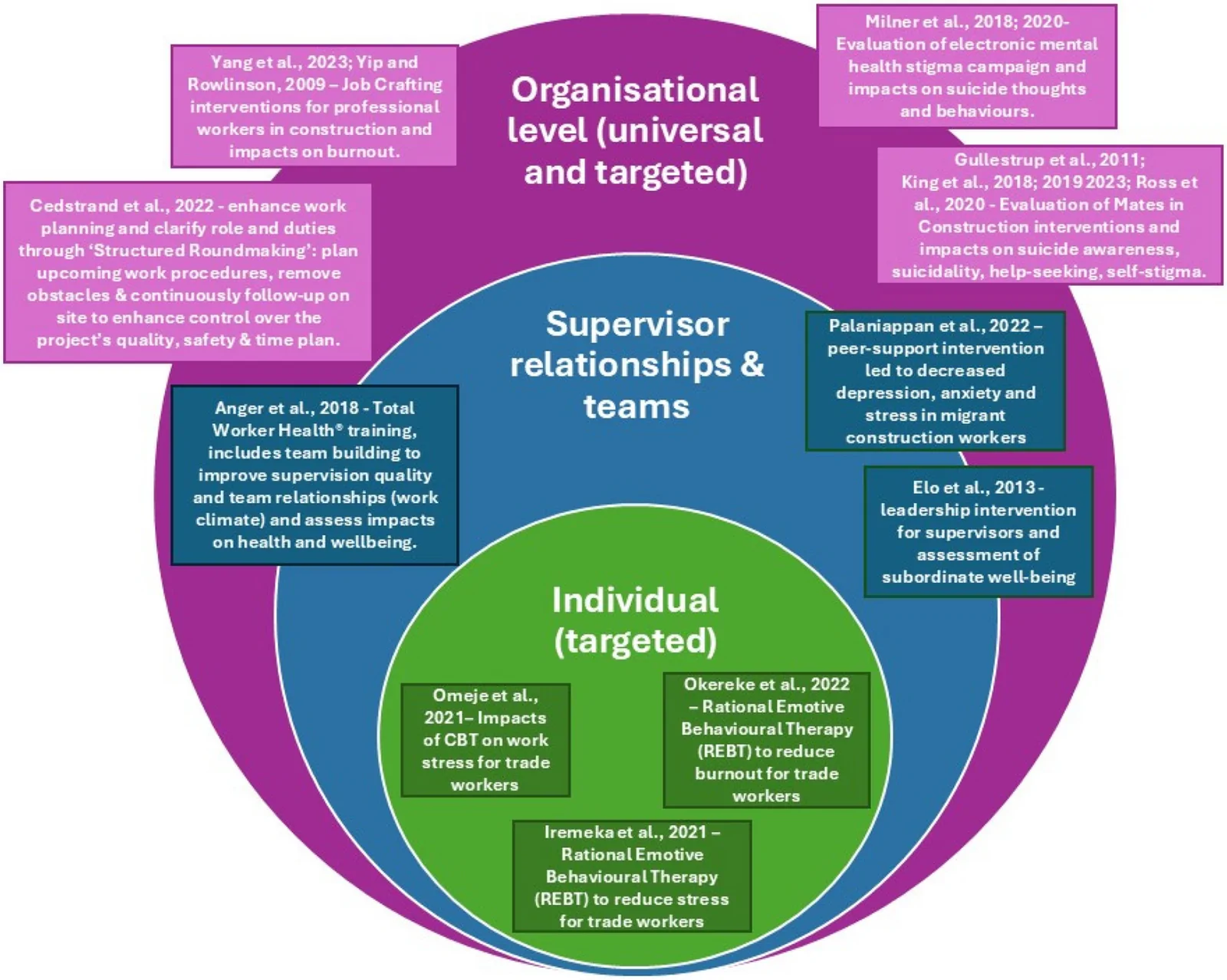
Graphical summary of the 16 interventions by type: organisational, supervisor relationships and teams and individual (targeted)

Ten of the studies tested organisational level interventions that were either targeted or universal. Two of these studies tested job crafting interventions to reduce worker burnout [6, 45]. Five studies evaluated components of the Australian ‘Mates in Construction (MATES)’ multimodal workplace focussed suicide prevention programme[49, 50, 55–57]. Two further studies [51, 52] evaluated Contact + Connect, an Australian electronic mental health stigma intervention. One study attempted to address job control and role clarity through a co-created intervention to enhance planning, role and duties clarification and assess impacts on stress and psychosocial conditions [47]. Three interventions attempted to improve relationships with supervisors and peers through supervisor- or peer-training [46, 48, 60]. Three interventions targeted individuals testing the impacts of psychological therapies on stress and metal health symptoms [53, 58, 59]. All interventions are summarised in Table 2, Fig. 2 and study design and participant characteristics can be found in supplementary file 2.

### Which outcomes were reported?

A total of 43 mental wellbeing relevant outcomes were reported across the 16 included studies (Fig. 2). Each study reported between 1 and 10 such outcomes. There was little consistency in outcome reporting with most outcomes (n = 39/43; 90.7%) reported just once across all reviewed studies. Outcomes that were reported two times or more, included: burnout, mental health, perceived stress, stress, help seeking intentions, suicide and suicide prevention awareness and beliefs, suicide prevention literacy and psychosocial safety climate. None of the studies reported economic outcomes.

In total, 23 clearly defined Outcome Measurement Instruments (OMIs) were utilised to measure 34/43 of the outcomes. The outcomes where there was some consistency i.e., where an OMI was used in two studies or more included: burnout, measured using Maslach’s burnout inventory [61, 62] in 3 studies [6, 45] stress measured using the Copenhagen Psychosocial Questionnaire III [63, 64] in two studies [46, 47], and finally, psychosocial safety climate measured using the Psychosocial Safety Climate Questionnaire [65] in two studies [46, 47].

For 11 of the 43 outcomes, new OMIs were proposed by study authors (Table 3); all were self-report questionnaires except one study where the number of calls made to an emergency helpline (and the number of calls requested post intervention) were captured to assess help-seeking behaviour.

**Table 3:** Outcome Domain Mapping and Outcome Measurement Instruments (OMIs)

Inductive categorisation		Extracted information		
**Outcome Domain**	**Sub-domain**	**Outcome**	**Number of times reported (reference)**	**Outcome Measurement Instrument (OMI) used to measure outcome**
(1) Mental health and wellbeing	Mental health	Anxiety	1 (Palaniappan 2022) [60]	Depression Anxiety Stress Scales—Self Report—21 questions questionnaire (DASS-21) [66]
		Burnout	4 (Elo 2014; Okereke 2022; Yang 2023; Yip 2009) [6, 45, 48, 59]	The Copenhagen Burnout Inventory [67] Depression Anxiety Stress Scales—Self Report—21 questions questionnaire (DASS-21) [66]
				Maslach's Burnout Inventory [61] – Chinese version for one study (Yang et al., 2003)
		Mental health	3 (Anger 2018; Palaniappan 2022) [46, 60]	5-item subset of the 20-item Centre for Epidemiological Studies Depression Scale [68]
				12-Item Short Form Health Survey (SF-12) [69]
				Depression Anxiety Stress Scales - Self Report - 21 questions questionnaire (DASS-21) [66]
	Stress	Occupational stress	1 (Omeje 2021) [53]	New OMI developed by the study authors (Artisans Occupational Stress Questionnaire)
		Occupational stress – work family conflict	1 (Anger 2018) [46]	Work–family conflict scale [70]
		Occupational stress – Family work conflict	1 (Anger 2018) [46]	Family–work conflict scale [70]
		Perceived stress	2 (Elo 2014; Iremeka 2021) [48, 58]	Perceived Stress Scale—14 (PSS-14) [71]
				Single item perceived stress scale [72]
		Stress	2 (Cedstrand 2022; Palaniappan 2022) [47, 54]	Copenhagen Psychosocial Questionnaire III (COPSOQ) [63]
				Depression Anxiety Stress Scales—Self Report—21 questions questionnaire (DASS-21) [66]
	Life- and work-related wellbeing	Work related irrational beliefs	1 (Iremeka 2021) [58]	Work Related Irrational Beliefs Scale (WIB-Q) [73]
		Dysfunctional thoughts at work	1 (Omeje 2021) [53]	New OMI developed by study authors (Artisans’ dysfunctional thoughts at work questionnaire)
		Job satisfaction	1 (Anger 2018) [46]	The Michigan Organizational Assessment Questionnaire -Job Satisfaction Subscale [74]
		Emotional wellbeing	1 (Ross 2020) [57]	OMI developed by researchers
		Life satisfaction	1 (Anger 2018) [46]	The Satisfaction with Life Scale [75]
(2) Work processes & environment	Work relationships	Feedback from supervisor	1 (Elo 2014) [48]	The Healthy Organisations Questionnaire [76]
		Support from supervisor	1 (Elo 2014) [48]	The Healthy Organisations Questionnaire [76]
		Justice of leadership	1 (Elo 2014) [48]	The Healthy Organisations Questionnaire [76]
		Team Effectiveness	1 (Cedstrand 2022) [47]	Team Effectiveness Scale [77]
		Team cohesion	1 (Anger 2018) [46]	Perceived Cohesion Scale [78]
		Work climate	1 (Elo 2014) [48]	The Healthy Organisations questionnaire [76]
		Family-supportive supervisor behaviours – supervisor	1 (Elo 2014) [48]	Family Supportive Supervisor Behaviour Short-Form [79]
		Family-supportive supervisor behaviours – employee	1 (Anger 2018) [46]	Family Supportive Supervisor Behaviours [80]
	Organisational processes	Flow of information at the workplace	1 (Elo 2014) [48]	The Healthy Organisations Questionnaire [76]
		Planning	1 (Cedstrand 2022) [47]	Name of measure unclear & non-English [81]
		Staffing	1 (Cedstrand 2022) [47]	Name of measure unclear & non-English [81]
	Workplace psychosocial hazards	Job demands	1 (Elo 2014) [48]	The Healthy Organisations Questionnaire [76]
		Job control	1 (Elo 2014) [48]	The Healthy Organisations Questionnaire [76]
		Psychosocial Safety Climate	2 (Anger 2018; Cedstrand 2022) [46, 47]	Name of measure unclear [82] The Psychosocial Safety Climate Questionnaire [65]
		Quantitative Demands	1 (Cedstrand 2022) [47]	Copenhagen Psychosocial Questionnaire III (COPSOQ) [63]
		Role Clarity	1 (Cedstrand 2022) [47]	Copenhagen Psychosocial Questionnaire III (COPSOQ) [63]
(3) Mental health stigma, behaviours or attitudes	Internalised stigma	Self-blame	1 (Milner 2018) [51]	The Self-stigma of Depression Scale (SSDS) (Self-blame subscale) [83]
		Shame	1 (Milner 2018) [51]	The Self-stigma of Depression Scale (SSDS) (Shame subscale) [83]
	Stigma related behaviours	Help seeking inhibition	1 (Milner 2018) [51]	The Self-stigma of Depression Scale (SSDS) (Help seeking subscale) [83]
		Help seeking and help offering propensity	1 (King 2018) [50]	New OMI developed by study authors
		Help-seeking intentions	2 (King 2023; Ross 2020) [49, 57]	Adapted version of the General Help-Seeking Questionnaire (adapted by the study authors) [84]
		Help-seeking behaviour	1 (Gullestrup 2011) [55]	Number of calls made to emergency helpline and number of follow up calls requested
(4) Suicide awareness, knowledge, beliefs or behaviours	Suicide knowledge, awareness, or beliefs	Suicide and suicide prevention awareness and beliefs	2 (Gullestrup 2011; King 2019) [55, 56]	Participants rate agreement with common suicide myths – assessment developed by study authors
		Suicide prevention literacy	2 (King 2023; King 2019) [49, 56]	Participants rate agreement with common suicide myths – assessment developed by study authors
		Suicide awareness	1 (Ross 2020) [57]	New OMI developed by study authors
		Attitudes to the workplace in preventing suicide	1 (King 2019) [56]	New OMI developed by study authors (adapted from their own prior work)
		Suicidal ideation	1 (Milner 2020) [52]	The Suicidal Behaviours Questionnaire Revised [85]
	Suicide related behaviours or experiences	Communication about suicide	1 (Milner 2020) [52]	The Suicidal Behaviours Questionnaire Revised [85]
		Experiences of Suicide and helping behaviours	1 (King, 2018) [50]	OMI developed by researchers
		Suicide attempts	1 (Milner 2020) [52]	The suicidal Behaviours Questionnaire Revised [85]
4	10	43	54	

**Fig. 3:**
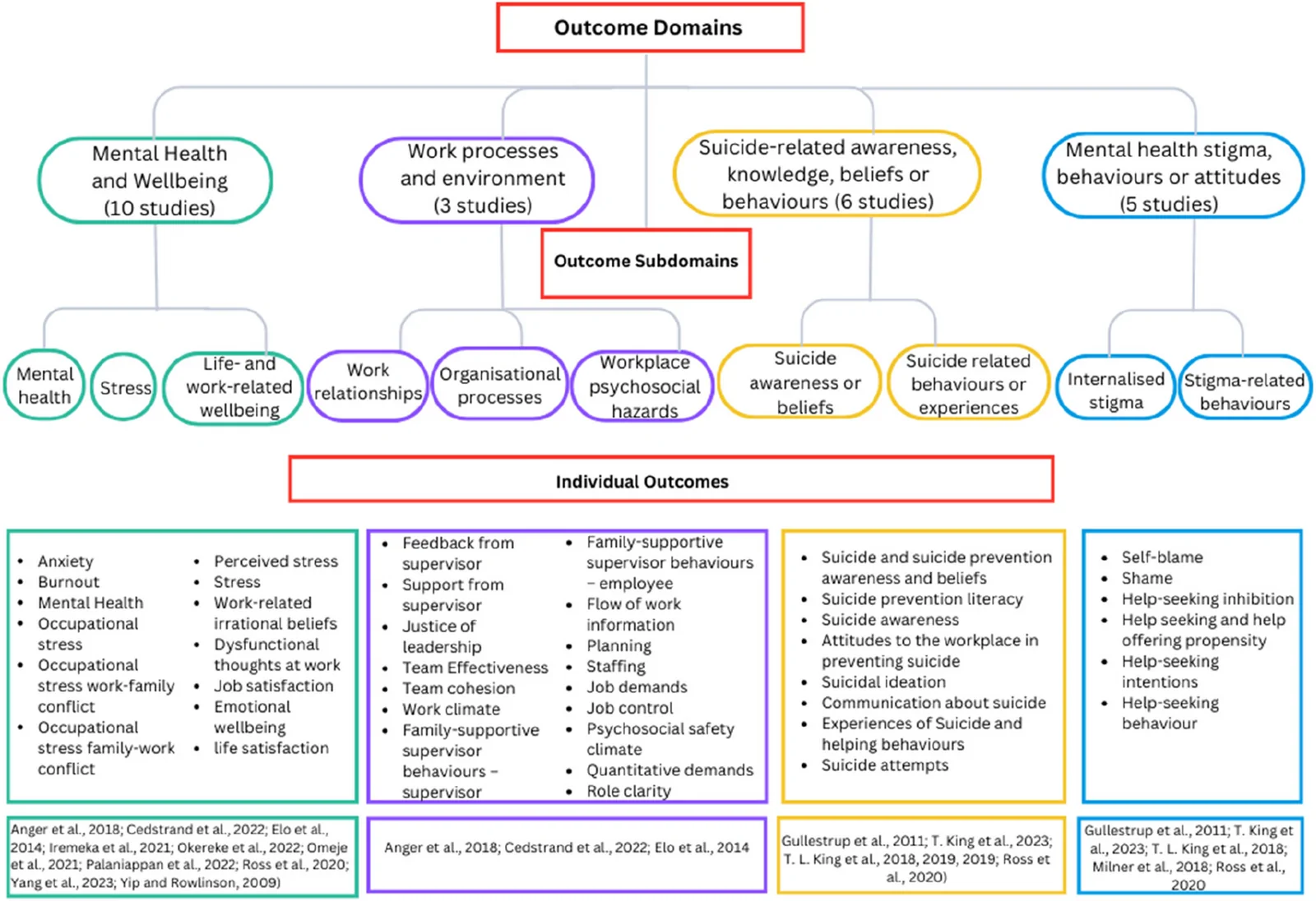
Outcomes measured in mental wellbeing intervention studies for construction workers

All the extracted outcomes were inductively assigned to four domains (Table 3 and Fig. 3): (1) Mental health and wellbeing, 2) Work processes and environment, (3) Mental health stigma, behaviours or attitudes, and (4) Suicide awareness, knowledge, beliefs or behaviours.

1. Mental health and wellbeing
  This definition emerged from inductive mapping and with reference to mental health definitions by [86]. This domain includes outcomes relating to workers’ mental health problems and consisted of 3 sub-domains; 1) mental health, 2) stress; and 3), life- and work-related wellbeing. Three outcomes belonged to the mental health subdomain, 5 to the stress subdomain and 5 to the life and work-related wellbeing subdomain.
  Of all the studies reviewed, this outcome domain was the most frequently reported by ten out of the 16 studies (62.5%) [46–48, 53, 57, 59, 60].
2. Work processes and environment
  This domain includes outcomes relating to factors within the work environment that could impact on workers’ mental wellbeing reported by three studies [46–48] all of which focused on changing elements of the work environment (i.e., environmental, relational and organisational) through supervisor or peer training to improve mental wellbeing.
  The Work Environment domain consists of 3 sub-domains; 1) work environment and relationships; 2) organisational processes and 3) workplace psychosocial hazards.
  In total 16/43 (37.2%) of the outcomes belonged to this domain. The work environment and relationships subdomain contained eight outcomes, the organisational processes subdomains included three outcomes, and the workplace psychosocial hazards subdomain had five outcomes.
3. Mental health stigma, behaviours, or attitudes
  This domain includes outcomes relating to mental health related stigma and attitudes and consisted of two sub-domains; 1) internalised stigma; and 2) stigma-related behaviours.
  In total 6/43 (14%) of the outcomes belonged to this domain. In terms of the subdomains, 2 outcomes belonged to the internalised stigma subdomain and 4 outcomes belonged to the stigma related behaviours subdomain. Five studies reported outcomes belonging to this domain [49–51, 55, 57]. Notably, these were all studies evaluating either the Australian MATES multimodal workplace focussed suicide prevention programme or the Contact + Connect intervention.
4. Suicide awareness, knowledge, beliefs, or behaviours
  This domain includes outcomes relating to suicide and consisted of 2 sub-domains; 1) Suicide knowledge, awareness, or beliefs; and 2) Suicide related behaviours or experiences.
  In total 8/45 (17.78%) of the outcomes belonged to this domain. Five of the outcomes belonged to the suicide knowledge, awareness, or beliefs sub domain and 3 outcomes were categorised to the suicide related behaviours of experiences subdomain. Six studies (n = 6/14; 42.86%) had outcomes belonging to this domain [49, 50, 55, 56, 56, 57]. Again, these were all the studies evaluating the Australian MATES workplace focussed suicide prevention programme or the Contact + Connect interventions.

### Timing of assessment

Seven studies reported outcome data pre and immediately post intervention [6, 45, 46, 50–52, 56]. Nine studies collected and reported follow up outcome data at time points ranging from 3 to 24 months [45–49, 53, 54, 57, 58]. However, for one study [59] the specific timing of follow up assessments was unclear.

## Discussion

This review summarises interventions studies to improve the mental wellbeing of construction workers captured using a search strategy based on intervention types summarised in the NICE Mental Wellbeing guidance (2022) [41]. Considerable heterogeneity in outcome reporting across intervention studies within the construction industry was identified, thus justifying the need for a COS to offer practitioners better guidance for improving the management of mental wellbeing in construction workers. Of the four outcome domains identified, the greatest number of outcomes were reported within the ‘work processes and environment’ domain. To our knowledge, this is the first study of outcome reporting for mental wellbeing at work interventions. Although many COS from health sciences include mainly health-related outcomes, this observation highlights a potential distinction; that is that factors in the work environment (subdomains: relationships, processes and psychosocial hazards) are also important. This observation falls in line with evidence that work processes and environment negatively impacts mental wellbeing for construction workers [47, 48]. Unexpectedly, none of the studies reported economic outcomes relevant to productivity, or return on investment; nor were financial stressors for workers explored in spite the literature suggesting financial and job insecurity as common psychosocial hazards [13].

Reflecting the commonly observed discourse around stoicism and a reluctance to engage in help seeking behaviours within the construction industry [87, 88], five studies reported on outcomes relating to mental health stigma, behaviours or attitudes [49–51, 55, 57]. Likewise, in line with the disproportionately high suicide rates among construction workers [8, 11], six studies had outcomes belonging to the suicide awareness, knowledge, beliefs or behaviours domain [49, 50, 55, 56, 56, 57]. All of the studies reporting on outcomes belonging to the latter two domains were evaluating either the MATES multimodal workplace focussed suicide prevention programme or the Contact + Connect intervention.

Prior reviews evaluating the effectiveness of workplace interventions, including in the construction industry, have noted a deficit in the reporting of positive mental wellbeing outcomes and instead a focus on pathology-oriented concepts of mental health such as burnout and stress [43, 89]. Supporting this observation, within the current review, the mental health and wellbeing domain comprised of mostly negatively framed aspects of wellbeing accounting for 11/15 of the outcomes within this domain, whereas positive mental wellbeing outcomes (e.g., emotional wellbeing, life satisfaction, wellbeing, and job satisfaction) accounted for only 4/15 outcomes. Wellbeing is often conceptualised as a continuum from psychological ill-being, such as burnout and stress, to facets of positive well-being, such as happiness, vitality, or sense of purpose [89]. Therefore, a dominance of pathology-orientated outcomes could limit our understanding of interventions that can promote healthy mental functioning in construction workers. The development of a COS and involvement of a COS working group would enable discussion and consideration of calls from previous systematic reviews such as Greiner et al., [43] for the utilisation of a greater range of mental health and wellbeing outcomes when evaluating workplace interventions. By identifying and standardising a minimum set of relevant and meaningful outcomes (including aspects of positive mental wellbeing), a COS can help to ensure that all key aspects of mental health and wellbeing are captured across all studies. Accordingly, COS development may facilitate the expansion of the outcome landscape beyond negatively oriented aspects of wellbeing indicators, supporting a more comprehensive understanding of mental health and wellbeing within the construction industry.

This review emphasises that reporting outcomes relating to work processes and environment is common and most probably reflects the authors’ interest in measuring the predictors of poor mental wellbeing in intervention studies. This falls in line with organisational interventions which are often defined as targeting the psychosocial work environment [43]. Such approaches offer the potential to address underlying work-related risk factors for poor mental wellbeing which are prevalent within the sector (e.g., low job control, high job demand, poor working conditions) rather than placing sole responsibility on the individual worker for their wellbeing [90, 91]. It is therefore important for these interventions to also assess effectiveness based on organisational/group level change as these may be an intermediary outcome that can improve worker wellbeing [43, 92]. Consequently, we felt it important to capture outcomes relating to this domain, so that this information can be fed into a future COS development process. Measurement of work environment variables alongside individual level wellbeing variables could facilitate our knowledge of work characteristics that could be targeted by future interventions to improve construction worker wellbeing [43].

### Strengths and limitations

To the authors’ knowledge, this is the first systematic review to investigate outcome reporting trends within mental wellbeing interventions for any industry. We chose to focus on the construction industry and reveal novel insights regarding the heterogeneity of reporting. Strengths of this review include its systematic and comprehensive search strategy, in addition to its broad criteria in terms of study design inclusion criteria which ensured we could capture all intervention studies within the topic scope and were not restricted to those with an RCT design. Furthermore, although only English language publications were included, international publications were viable for inclusion, reflecting the international focus of a COS. As we focused on reported outcomes, we acknowledge that this may not be reflective of what was actually measured within the studies, i.e., we did not examine reporting bias as proposed by some for COS systematic reviews [93, 94]. Finally, although we identified and reported all Outcome Measurement Instruments (OMIs), we did not assess the relative quality (that is, evidence of essential measurement properties) nor acceptability as this was beyond the scope of this review but would be an important consideration in later stages of the COS development.

### Recommendations for future research

This review supports the NICE NG212 research recommendation to develop a COS for mental wellbeing interventions [95]. We have approached this using an industry-specific approach based on distinctive occupational challenges faced by construction workers [3, 13, 14, 25], and the urgent need to support their wellbeing [26, 27]. The findings from this systematic review will contribute to the initial phases of COS development according to international guidance [96]. In particular, consideration of the scope of the COS by a Working Group of experts and identification of other areas where reviews of outcomes or factors used in previous studies are needed prior to multistakeholder prioritisation and consensus [97]. For the construction industry, development of a COS, co-created with construction workers and other stakeholder groups could provide standardisation of outcome assessment for future research and intervention studies relevant for researchers, industry bodies, workplace health and wellbeing and public health practitioners. It would also ensure the relevance of outcomes to all stakeholders, facilitate comparability between studies and reduce research wastage [30–32, 94, 98].

Development of a new COS, as recommended in COS [99] requires consideration of differential experiences of the workforce. This is important for a construction industry-relevant COS, especially since subgroup analysis shows that professional versus manual workers are differentially impact by psychosocial stressors [17]. As construction is male-dominated, previous research points to women within construction experiencing unique stressors including benevolent and hostile sexism [100–102]. There is also evidence that migrant workers are more vulnerable to discrimination, language barriers, time pressures and poor working environments [103]. In many countries, a significant proportion of the construction workforce are self-employed [18, 22] working without statutory employment rights, a risk factor of poor mental wellbeing in itself [104]. Having extracted data on characteristics of workers as participants in the studies included in this review, we found that 12 of 16 studies failed to report the ethnic origin of their sample (see Supplementary file 2). Furthermore, 4 studies did not report gender, and 3 studies did not report the age of participants. This lack of detail restricts the ability to determine whether the outcomes used in prior intervention studies have been applied in diverse populations and highlights the need for improved demographic reporting in intervention studies to support more equitable and generalisable research practices. Throughout the development of the COS, it will be crucial to incorporate the voices of all worker groups, particularly those from marginalised and/or vulnerable subgroups, to ensure that the COS is relevant across the workforce.

Finally, this review identified just 16 studies across all countries, evaluating ten discrete mental wellbeing interventions for construction workers. Although only studies written in English and academic journals were included, this highlights a disconnect in the volume of literature describing psychosocial stressors and suicidality in the industry, compared to the number of intervention studies to improve mental wellbeing. Development and availability of a COS may encourage more research to develop and evaluate existing and new mental wellbeing interventions for this at-risk group of workers. Given the gap in reporting economic outcomes across these studies, inspiration for a future COS could draw on economic outcomes measured in intervention studies in other industries.

## Conclusion

Underpinned by evidence that the workplace and work can negatively impact mental wellbeing for construction workers, several interventions have been evaluated. Inconsistencies in outcome reporting revealed in this review may limit evidence synthesis and advancement in knowledge of the efficacy of mental wellbeing interventions for construction workers. A COS would offer the industry research-informed and co-created guidance on what to measure when assessing the effectiveness of mental wellbeing initiatives so that organisations can better understand what works and subsequently what to make available for their workforce. This review provides an important first step in the development of a COS which will act as a guide for industry and public health practitioners and researchers.

## Supplementary Information

Supplementary Material 1.

Supplementary Material 2.

Supplementary Material 3.

Supplementary Material 4.

## Data Availability

All data generated or analysed during this study are included in this published article [and its supplementary information files].
