# Supplementary material for: Workplace mental wellbeing interventions in the construction industry: a systematic review of outcome reporting as a first step for the development of a core outcomes set
Source: BMC Public Health. 2026 Jul 16;26:2933. doi: 10.1186/s12889-026-28427-5 (PMC13656663; doi:10.1186/s12889-026-28427-5)
Supplement: Supplementary file 1 [file 12889_2026_28427_MOESM1_ESM.docx]

Supplementary File 1: Search Terms

| **Topic** | **Terms** |
| --- | --- |
| Construction workforce | "construction industr*" OR "construction sector" OR "construction trade" OR "construction compan*" OR "construction worker*" OR "trade worker*" OR "manual worker*" OR labourer* OR laborer OR "blue collar worker*" |
| Wellbeing | Wellbeing OR well-being OR "mental health" OR "psychological health" OR "psychological well-being" OR "mental well-being" OR "mental wellbeing" OR "mental wellness" OR stress OR burnout OR "workplace stress" OR "job stress" OR "occupational stress" OR anxiety OR depression |
| Intervention | Intervention* OR initiative* OR program* OR prevention OR training OR policy OR promotion OR campaign* |
| Workplace | Work OR workplace OR "work place" OR organisation* OR organization* |
