# Supplementary material for: Workplace mental wellbeing interventions in the construction industry: a systematic review of outcome reporting as a first step for the development of a core outcomes set
Source: BMC Public Health. 2026 Jul 16;26:2933. doi: 10.1186/s12889-026-28427-5 (PMC13656663; doi:10.1186/s12889-026-28427-5)
Supplement: Supplementary file 2 [file 12889_2026_28427_MOESM2_ESM.docx]

| **Supplementary file 2: Intervention study design and participant characteristics table**  ***Intervention study design and participant characteristics*** | | | | | | | |
| --- | --- | --- | --- | --- | --- | --- | --- |
| **First Author and year** | **Country** | **Type of Study/ design** | **Participant occupation** | **Intervention Group** | | **Control Group** | |
|  |  |  |  | **Sample size (% male)** | **Age**  **(Mean or %)** | **Sample size (% male)** | **Age (M/ %)** |
| Anger 2018 (46) | United States | Uncontrolled before and after study (pilot) | Supervisors and direct reports (with professional or trade roles) ** | Supervisors: 22 (90.9)  Employees: 13 (69.2) | Supervisors:  39.2  Employees: 37 | N/A | N/A |
| Cedstrand 2022  (47) | Sweden | Non- randomised controlled trial | Trade workers and professionals * | Baseline: 203 (79.4%)  12 month follow up: 189  24 month follow up: 176  Complete cases: 101 | 39.4 | Baseline: 124 (83.9%)  12 month follow up: 135  24 month follow up: 104  Complete cases: 41 | 44.2 |
| Elo 2014 (48) | Finland | Non-randomised, controlled before and after study | Supervisors and direct reports (trade workers and professionals * | Subordinates (working for 8 supervisors that took part in the intervention): 49 (67%) | 44.7 | Subordinates (working for 32 supervisors who did not take part in the intervention: 96 (84%) | 43.9 |
| Gullestrup 2011 (55) | Australia | Non-equivalent group comparison | Occupation not reported (Recruited from public and private sector construction sites) * | 7311 (gender not reported) | Not reported | 355 (gender not reported) | Not reported |
| Iremeka 2021 (58) | Nigeria | Randomised controlled trial | Skilled construction workers ** | 80 (80%) | Not reported | 80 (75%) | Not reported |
| King 2018 (50) | Australia | Uncontrolled before and after study | Technicians, trade workers. Labourers, machinery operators, managers, professionals and clerical/admin workers * | 20, 125 (92.5%) | 15-24 years: 14.8%  25-34 years: 32%  35+ years: 53.2% | N/A | N/A |
| King 2019 (56) | Australia | Uncontrolled before and after study | Technicians, trade workers. Labourers, machinery operators, managers, professionals and clerical/admin workers * | 19,917 | 15-24 years: 14.9%  25-34 years: 31.6%  35-44 years: 23.9%  45+ years: 29.6% | N/A | N/A |
| King 2023 (49) | Australia | Randomised controlled trial | Trade workers and professionals ** | Baseline: 509 (88.8%)  Received allocated intervention: 86  Included in final analysis: 509 | 17-29 years: 28.3%  30-39 years: 23.2%  40+ years: 38.5%  Missing: 10% | Baseline: 575 (84.9%)  Included in final analysis: 575 | 17-29 years: 27.8%  30-39 years: 25.2%  40-49 years: 19.3%  50-59 years: 11.8%  60+ years: 4.5%  Missing: 11.3% |
| Milner 2018 (51) | Australia | Randomised controlled trial | Occupation not reported * | Baseline: 343 (100%)  Follow up: 247  (100%) | 18-29 years: 9.8%  30-39 years: 22.1%  40+ years: 68% | Baseline: 302 (100%)  Follow up: 231  (100%) | 18-29 years: 11.4%  30-39 years: 25.8%  40-49 years: 31.8%  50-59 years: 22.9%  60 years plus: 8.1% |
| Milner 2020 (52) | Australia | Randomised controlled trial | Occupation not reported * | Baseline: 343 (100%)  Follow up: 243 | 18-29 years: 11.1%  30-39 years: 21.8%  40+ years: 67% | Baseline: 302 (100%)  Follow up: 225 | 18-29 years: 11.1%  30-39 years: 24.9%  40-49 years: 32.9%  50-59 years: 21.8%  60+ years: 9.3% |
| Okereke 2021 (59) | Nigeria | Randomised controlled trial | Trades (incl. masonry/bricklaying, carpentry, plumbing, tiling) * | 63 (gender not reported) | 40.70 | 62 (gender not reported) | 42.16 |
| Omeje 2021 (46) | Nigeria | Randomised controlled trial | Trade workers (e.g., carpenters, electricians, tilers) * | 70 | 20-30 years: 30%  31-40 years: 27.1%  41-45 years: 21.4%  45+ years: 21.4% | 70 | 20-30 years: 30%  31-40 years: 24.3%  41-45 years: 21.4%  45+ years: 24.3% |
| Palaniappan 2022 (60) | Singapore | Uncontrolled before and after and follow up study | Occupation not reported ** | 348 (99.1%) | <25 years: 18.4%  26-25 years: 46%  36-45 years: 23%  >45 years: 12.9 | N/A | N/A |
| Ross 2020 (57) | Australia | Before, after follow-up Comparison study | Occupation not reported * | GAT:  Baseline: 2260 (91.8%)  Post: 2241 (91.8%)  Follow up: 189 (83.4%)  MAT:  Baseline: 717 (95.5%)  Post: 700 (95.9%)  Follow up: 56 (91.1%) | GAT:  Baseline: 34.94  Post: 34.89  Follow-up: 41.73  MAT:  Baseline: 30.56  Post: 30.43  Follow up: 37.43 | N/A | N/A |
| Yang 2023 (44) | China | Randomised controlled trial | Professional (project managers) * | 36 (89%) | 21 to 25 (49%) 26 to 30 (20%) 31 to 35 (31%) | 36 (89%) | 21 to 25 (29%)  26 to 30 (34%)  31 to 35 (37%) |
| Yip 2009 (6) | Hong Kong | Uncontrolled before and after study | Professional * | 55 completed follow-up after 1 year (gender not reported) | Age not reported |  |  |

***Key: * Ethnic origin of participants was not reported;*** ** ***Ethnic origin of participants was reported***
